# Inhibitive Properties of Benzyldimethyldodecylammonium Chloride on Microbial Corrosion of 304 Stainless Steel in a *Desulfovibrio desulfuricans*-Inoculated Medium

**DOI:** 10.3390/ma12020307

**Published:** 2019-01-18

**Authors:** Chung-Wen Hsu, Tzu-En Chen, Kai-Yin Lo, Yueh-Lien Lee

**Affiliations:** 1 Department of Engineering Science and Ocean Engineering, National Taiwan University, Taipei 106, Taiwan; r05525021@ntu.edu.tw (C.-W.H.); r07525016@ntu.edu.tw (T.-E.C.); 2 Department of Agricultural Chemistry, National Taiwan University, Taipei 106, Taiwan; kaiyin@ntu.edu.tw

**Keywords:** microbial corrosion, sulfate-reducing bacteria, biocides, electrochemical test, confocal laser scanning microscopy

## Abstract

Biocides are frequently used to control sulfate-reducing bacteria (SRB) in biofouling. The increasing restrictions of environmental regulations and growing safety concerns on the use of biocides result in efforts to minimize the amount of biocide use and develop environmentally friendly biocides. In this study, the antimicrobial activity and corrosion inhibition effect of a low-toxic alternative biocide, benzyldimethyldodecylammonium chloride (BDMDAC), on a 304 stainless steel substrate immersed in a *Desulfovibrio desulfuricans* (*D. desulfuricans*)-inoculated medium was examined. Potentiodynamic polarization curves were used to analyze corrosion behavior. Biofilm formation and corrosion products on the surfaces of 304 stainless steel coupons were examined using scanning electron microscopy (SEM), energy-dispersive X-ray spectrum, and confocal laser scanning microscopy (CLSM). Results demonstrated that this compound exhibited satisfactory results against microbial corrosion by *D. desulfuricans*. The corrosion current density and current densities in the anodic region were lower in the presence of BDMDAC in the *D. desulfuricans*-inoculated medium. SEM and CLSM analyses revealed that the presence of BDMDAC mitigated formation of biofilm by *D. desulfuricans*.

## 1. Introduction

Microbiologically influenced corrosion (MIC), which is caused by interactions between various microorganisms, is a long-term concern in many industries, including those involved with underground pipelines, storage vessels, shipping, and marine equipment. Reports indicate that MIC was responsible for at least 20% of all damaging corrosion, with a direct cost of $30–$50 billion annually worldwide [1,2]. Among the various types of micro-organisms, SRB are considered the major bacterial group responsible for microbial corrosion under anaerobic conditions [3,4]. Several studies have been conducted to investigate SRB-induced corrosion of iron substrate, and SRB are widely accepted to play a crucial role in the anaerobic MIC of iron, low-alloy steel, and stainless steel [5,6,7,8,9,10,11,12,13,14,15,16,17]. Various mechanisms explain the enhanced corrosion caused by SRB. Among these, the primary mechanism sees SRB reduce inorganic sulfate to hydrogen sulfide, resulting in the bulk equation (Fe + H_2_S → FeS + H_2_) [18,19,20,21]. Moreover, extracellular polymer substances and corrosive metabolites secreted by SRB [22,23], consumption of cathodic hydrogen and iron-derived electron transfers [24], and anodic depolarization resulting from the local acidification at the anode [25], all affect the corrosion behavior of iron.

Because of economic losses and safety hazards, it is important to control microbial corrosion by SRB and aggressive sulfide anions when they contact metal substrates. Several methods such as biocide treatment, cathodic protection, and addition of nitrate (or nitrite) have been developed to minimize the risks resulting from SRB activity [23,26]. Among these, biocide treatment is the most common method of controlling microbial corrosion. Organotins, such as dibutyltin and tributyltin (TBT), have been extensively used as antifouling agents in paints since the early 1970s. Despite their high resistance to microbial corrosion in marine environments, these compounds were banned in numerous parts of the world in the early 1990s due to the severe environmental and human health risks that they pose [27,28,29,30,31,32,33]. Therefore, nontoxic or less-toxic alternative biocides must be developed to substitute for TBT. 

Quaternary ammonium compounds (QACs) are considered candidates for biocides because of their excellent stability and antimicrobial properties [34,35,36]. QACs are surfactants with a hydrocarbon water-repellent (hydrophobic) group and a water-attracting group (hydrophilic). The antimicrobial properties of QACs mainly depend on the length of the long-chain alkyl group [37]. QACs have wide applications ranging from clinical to industrial purposes. For example, they are used for the disinfection of surfaces, equipment, and medical devices. Benzyldimethyldodecylammonium chloride (BDMDAC) is a quaternary ammonium compound with a C12-alkyl chain. The negative charge of a cell membrane easily attracts the positive charge of the ammonium group in BDMDAC. The hydrophobic C12-alkyl chain inserts into the membrane, causing disruption of bacterial cells [38]. Thus, BDMDAC is regarded as highly bactericidal. Comparing the median lethal dose (LD_50_) values of BDMDAC with the TBT obtained from their respective material safety datasheets (Alfa Aesar, Ward Hill, MA, USA, 2015), the LD_50_ value for BDMDAC is two to three times higher than that of TBT (LD_50_ of BDMDAC: 400 mg/kg in rats; LD_50_ of TBT: 132 mg/kg in rats), suggesting BDMDAC is less toxic than TBT.

The objective of this study was to evaluate the antimicrobial properties of BDMDAC in anaerobic conditions by examining the effect of BDMDAC as a biocide on *D. desulfuricans*. The influence of BDMDAC and *D. desulfuricans* on the corrosion behavior of 304 stainless steel (304SS) was studied using polarization curves.

## 2. Materials and Methods

### 2.1. Sample Preparation

The 304SS coupons (2 × 1 × 0.1 cm^3^) with a testing area of 2 cm^2^ were prepared for the subsequent corrosion studies and biofilm observation. All the coupons were mechanically ground using emery papers of 200–1200 grit, rinsed with deionized water, and then washed with alcohol in an ultrasonic bath.

### 2.2. Bacteria and Culture Medium

*Desulfovibrio desulfuricans* subsp. *desulfuricans*, the SRB used in this study, was obtained from the Bioresource Collection and Research Center, Hsinchu, Taiwan. The medium used for *D. desulfuricans*, Postgate’s medium (DSMZ, *Desulfovibrio* medium, Medium 63), was prepared as follows [39,40,41]: A 980 mL solution A (0.5 g of K_2_HPO_4_, 1 g of NH_4_Cl, 2 g of MgSO_4_·7H_2_O, 1 g of Na_2_SO_4_, 0.1 g of CaCl_2_·2H_2_O, 1 g of yeast extract, and 2 g of sodium lactate) was boiled, and the dissolved oxygen in the solution was removed using a mechanical deaerator with nitrogen. A 10 mL solution B (0.1 g of ascorbic acid and 0.1 g of sodium thioglycolate) and a 10 mL solution C (0.5 g of FeSO_4_·7H_2_O) were added to solution A. The medium was adjusted to pH 7.8 with NaOH and autoclaved at 120 °C for 15 min. *D. desulfuricans* was inoculated in the medium and cultured at 37 °C.

### 2.3. Bactericidal Assay of BDMDAC

Optical density at 600 nm (OD_600_) was measured using UV/VIS spectrophotometer (Optizen Pop, Mecasys, Daejeon, Korea). The *D. desulfuricans* culture was left overnight and diluted to an OD_600_ of 0.1 with a fresh medium. Various concentrations of BDMDAC were added to the culture and the absorbance at 600 nm was recorded as bacterial growth curves. 

### 2.4. Electrochemical Measurements

Potentiodynamic polarization curves were deduced using a Gamry Reference 600 potentiostat (Warminster, PA, USA) to evaluate corrosion performance [5,6,8,42,43,44]. A standard three-electrode system comprising a graphite counter electrode and saturated calomel electrode (SCE) as a reference electrode was used in all electrochemical tests. Three polarization curve measurements were performed under different working solutions as follows: (1) 304SS coupons in a culture medium without *D. desulfuricans* inoculation and BDMDAC (labeled as a blank solution); (2) 304SS coupons in a *D. desulfuricans*-inoculated medium without BDMDAC (labeled as an SRB solution); and (3) 304SS coupons in a *D. desulfuricans*-inoculated medium with BDMDAC (labeled as a BDMDAC solution). Potentiodynamic polarization curve measurements were obtained by sweeping the potential from −0.5 to 1.5 V versus open circuit potential at a scan rate of 1 mV/s [42]. Corrosion potential (*E_corr_*) and current density (*I_corr_*) were determined through Tafel extrapolation. The test area on the 304SS coupons for all electrochemical tests was 2 cm^2^. 

### 2.5. Surface Characterization

Biofilm formation and corrosion products formed on the 304SS coupons after different immersion times in various working solutions were analyzed through scanning electron microscopy (SEM) (JSM-6510, JEOL, Tokyo, Japan) and energy-dispersive X-ray spectrum (EDS) (Inca x-act, Oxford Analytical Instruments, Abington, UK). After the immersion tests, the 304SS coupons were extracted, rinsed with distilled water, and then fixed with 2.5 wt % glutaraldehyde for 15 min, followed by dehydration in a graded series of ethanol solutions (30%, 50%, 70%, 90%, and 100% for 15 min each) and air drying [7,45,46,47]. The dried coupons were coated with plate platinum on the surface and then studied using SEM. EDS was performed to analyze the chemical compositions of the biofilms and corrosion products. The surface morphology of 304SS coupons after polarization curve measurements was examined under an optical microscope (OM) (SG-3006HM, SAGE Vision, New Taipei City, Taiwan).

### 2.6. CLSM

Confocal laser scanning microscopy (CLSM) (LSM780, Carl Zeiss, Jena, Germany) was used in this study to detect the live and dead cells in the biofilms [46,47,48,49]. The 304SS coupons were immersed in a culture medium without inoculation, or with *D. desulfuricans* inoculum in the absence or presence of 25 ppm BDMDAC at 37 °C at different time intervals up to 28 days. After cleaning the surface once with phosphate-buffered saline, the biofilms were stained with LIVE/DEAD^TM^ *Bac*Light^TM^ bacterial viability kit (L7012, Thermo Fisher Scientific, Waltham, MA, USA). After staining, the coupons were observed under a fluorescence microscope. Live cells and dead cells appeared green and red, respectively, in the biofilm. The three-dimensional (3D) scanning images obtained by CLSM were used to measure biofilm thicknesses [46,47].

## 3. Results and Discussion

### 3.1. Bactericidal Assay of BDMDAC 

**Figure 1:**
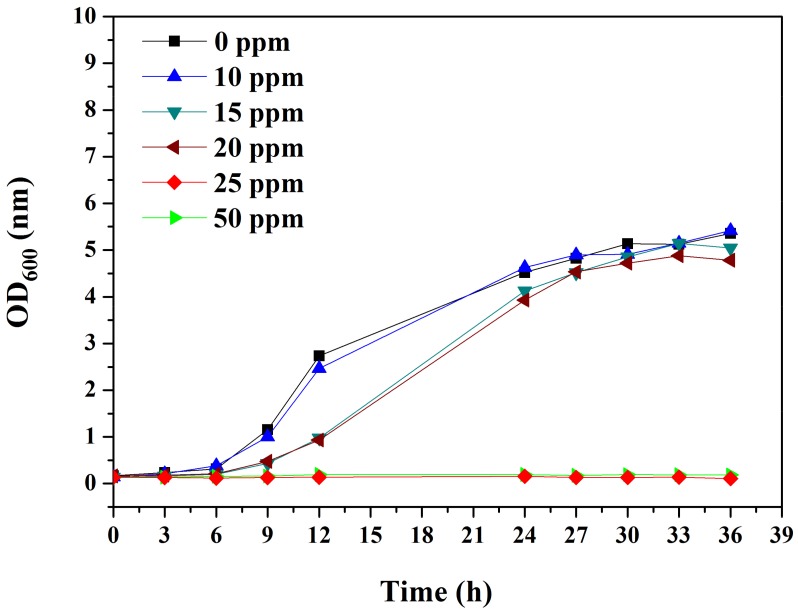
Effects of BDMDAC concentration on the growth curve of *D. desulfuricans* as a function of time in hours.

To test the antimicrobial activity of BDMDAC, the growth of *D. desulfuricans* was monitored in different concentrations of BDMDAC. The cell growth in 10 ppm BDMDAC did not differ from control (0 ppm) (Figure 1). When the BDMDAC concentration was elevated to 15 and 20 ppm, cell growth was slowed, with doubling time increasing from 12 to 18 h. Furthermore, when 25 and 50 ppm BDMDAC was added to the SRB solution, the cells did not grow. Therefore, the minimum inhibition concentration of BDMDAC for *D. desulfuricans* is 25 ppm.

**Figure 2:**
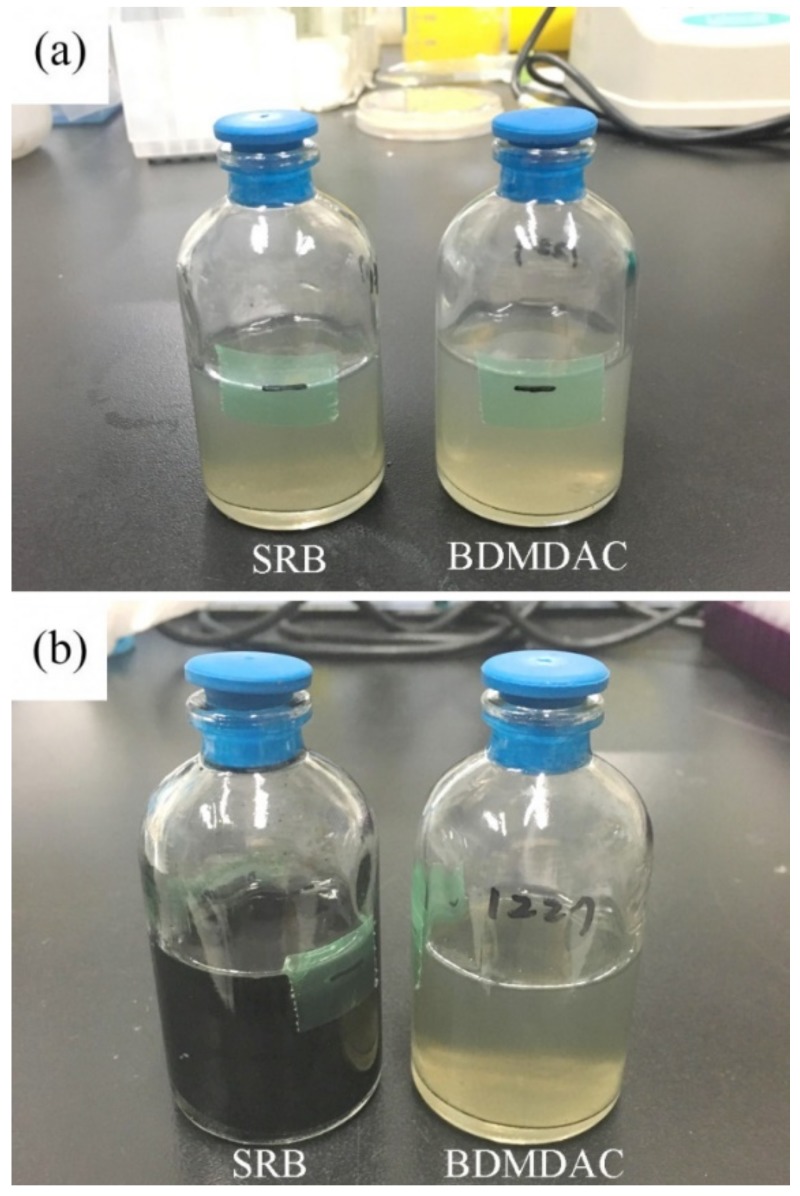
Visual inspection of the *D. desulfuricans*-inoculated medium with and without BDMDAC after (**a**) 7 days and (**b**) 28 days of incubation.

To validate the long-term antimicrobial effect, *D. desulfuricans* was inoculated in a medium containing 25 ppm BDMDAC, which was then incubated for 4 weeks at 37 °C. As shown in Figure 2, no growth was observed during this period. Thus, BDMDAC has a favorable inhibitory effect on the growth of *D. desulfuricans.*

### 3.2. SEM

**Figure 3:**
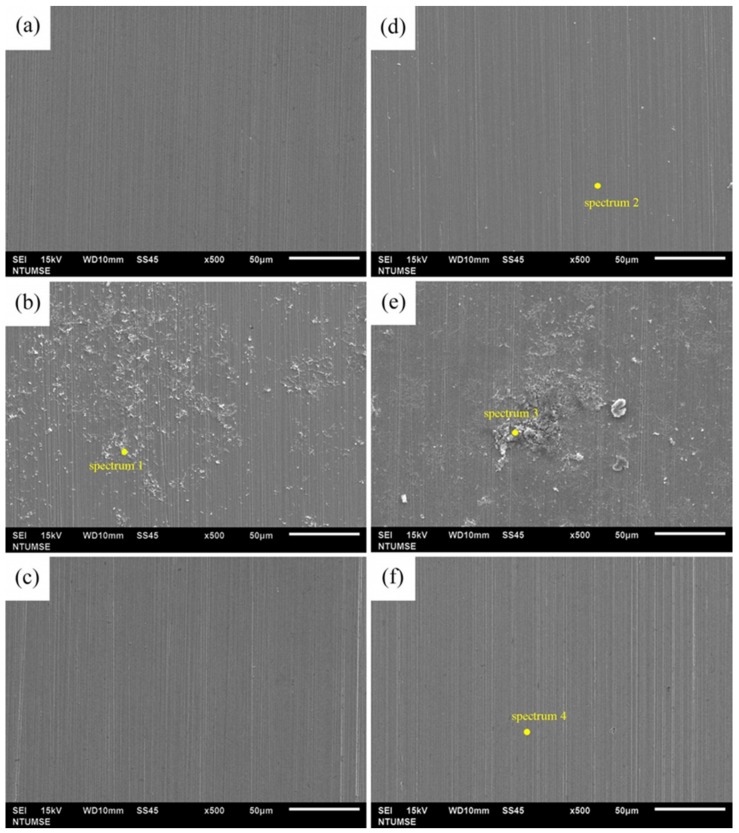
SEM images of the 304SS coupons after different immersion times in various solutions: (**a**) blank for 14 days; (**b**) SRB for 14 days; (**c**) BDMDAC for 14 days; (**d**) blank for 28 days; (**e**) SRB for 28 days; and (**f**) BDMDAC for 28 days.

Figure 3 displays the morphological characteristics of the biofilms grown on 304SS coupons after different immersion times in the *D. desulfuricans*-inoculated medium with and without BDMDAC addition. The surface morphologies of the 304SS coupons immersed in the culture medium without inoculation are shown for comparison. The SEM images reveal that the surfaces of the 304SS coupons were strongly influenced in the absence of *D. desulfuricans* and BDMDAC. First, Figure 3a,c show no visible corrosion products or biofilms formed on the 304SS coupons in the blank and BDMDAC solutions after 14 days of immersion, whereas Figure 3b displays corrosion products and biofilms on the 304SS coupon in the SRB solution. Some pitting is observable on the surface of the 304SS coupons in the blank and BDMDAC solutions, but this is only in a few locations. This observation is attributable to the presence of Cl^−^ ions in the culture medium [50,51,52]. The EDS elemental analysis results (Table 1) reveal that, in addition to characteristic corrosion products (Fe and O), sulfur elements were detected after 14 days of immersion in the SRB solution. Sulfur elements mainly result from the metabolic activity of SRB (formation of FeS) [53]. Thus, the EDS results suggest that the addition of BDMDAC in the SRB solution could decrease the activity of SRB and reduce the formation of biofilm. After extending the immersion time to 28 days, large areas of corrosion products and biofilms were observed on the coupons immersed in the SRB solution (Figure 3e), whereas the 304SS surface was largely intact in the blank and BDMDAC solutions (Figure 3d,f). Moreover, the EDS elemental analysis results revealed increased sulfur elements on the coupons immersed in the SRB solution. By contrast, sulfur elements were not observed on the 304SS coupons immersed in the blank and BDMDAC solutions. These results confirm the inhibition efficiency of 25 ppm BDMDAC.

### 3.3. CLSM

**Figure 4:**
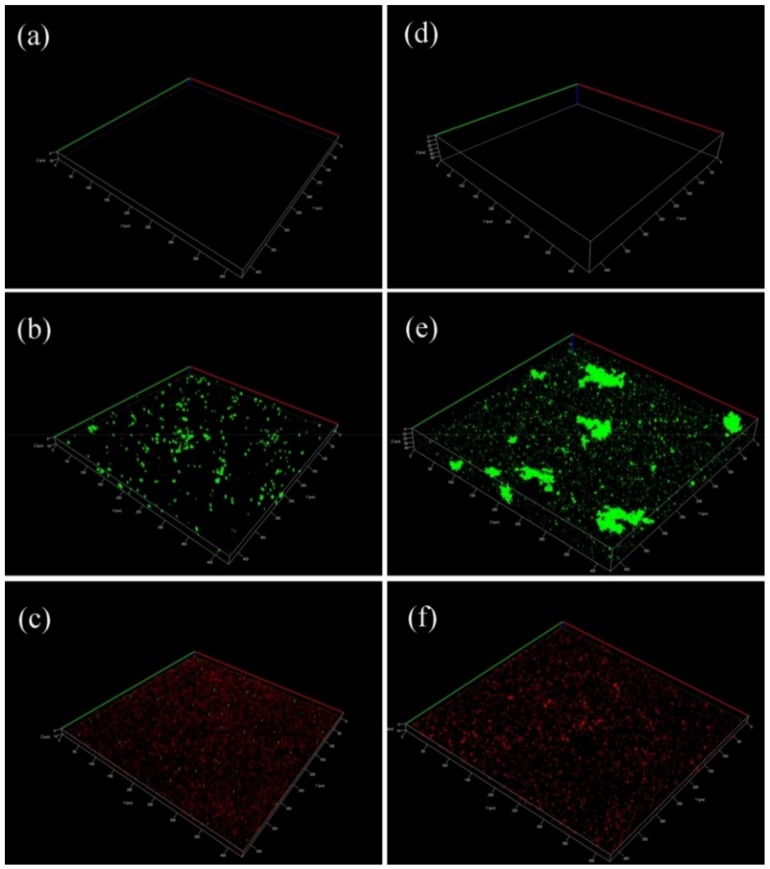
CLSM 3D images of the 304SS coupons after different immersion times in various solutions: (**a**) blank for 14 days; (**b**) SRB for 14 days; (**c**) BDMDAC for 14 days; (**d**) blank for 28 days; (**e**) SRB for 28 days; and (**f**) BDMDAC for 28 days.

To evaluate the inhibitory effect of BDMDAC to biofilm formation, CLSM was used for observation. Figure 4 shows the CLSM images of the 304SS coupons after 14 and 28 days of immersion in various solutions. In the culture medium without *D. desulfuricans* inoculation and BDMDAC, no growth was observed (Figure 4a,d). After incubation with *D. desulfuricans*, numerous green dots were observed, as shown in Figure 4b,e. The cells in the biofilm were stained green after staining, suggesting that most of the cells were alive. Furthermore, Figure 4b,e show that the thickness of biofilm increased to 18 and 40 µm for 14 and 28 days of immersion, respectively. In the prescence of BDMDAC, the thickness of biofilm was less than 20 µm, even after 4 weeks. Furthermore, most cells in the biofilm were now dead, as indicated by the red stains (Figure 4c,f). Notably, the biofilm grown on the 304SS coupons in the BDMDAC solution was not visible in the previous SEM images (Figure 3c,f). This can be attributed to the fact that killed bacteria or disrupted biofilm due to BDMDAC addition are easily removed during the cleaning process of SEM sample preparation.

### 3.4. Potentiodynamic Polarization

**Figure 5:**
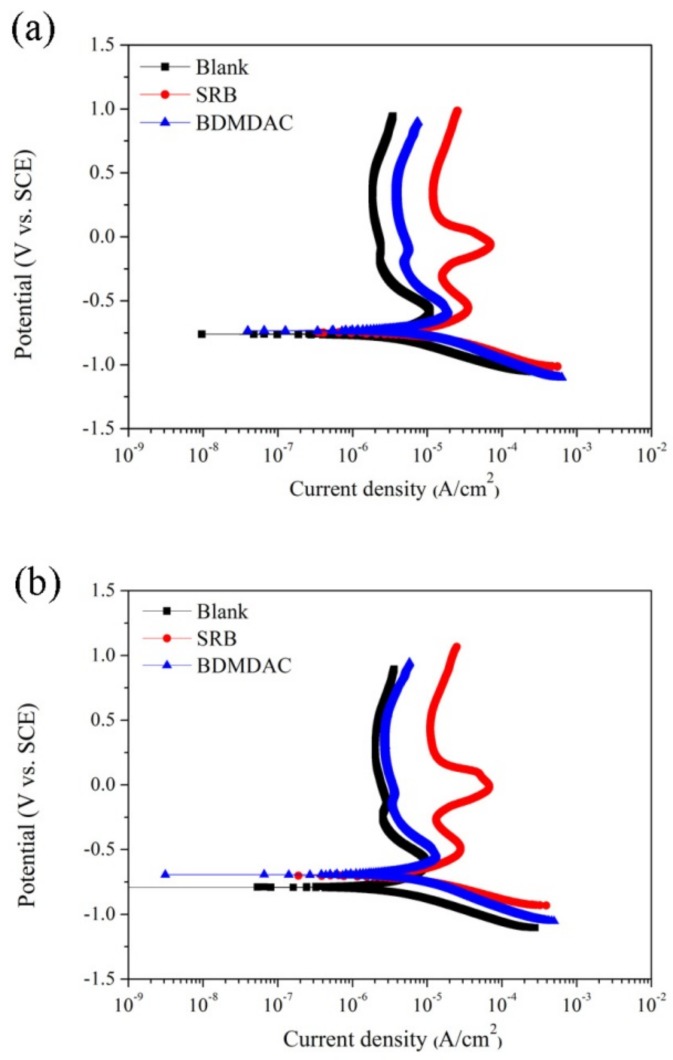
Potentiodynamic polarization curves for the 304SS coupons after 14 days (**a**) and 28 days (**b**) of immersion in various solutions.

**Figure 6:**
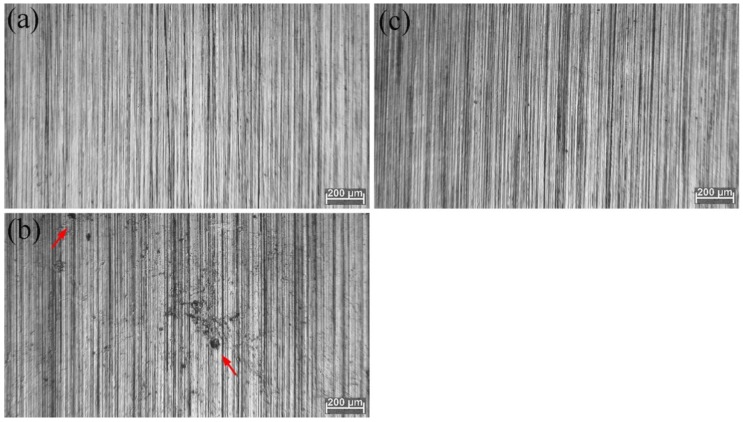
OM images of 304SS coupons after potentiodynamic polarization curve measurements. These surfaces were initially exposed to (**a**) blank for 14 days; (**b**) SRB for 14 days; and (**c**) BDMDAC for 14 days.

Figure 5 illustrates the potentiodynamic polarization curves of coupons under different immersion environments after 14- and 28-day immersion periods. The values of *E_corr_* and *I_corr_* according to the potentiodynamic polarization curve are listed in Table 2. Table 2 shows that the corrosion current densities *I_corr_* in the presence of bacteria were higher than that in the control medium for each corresponding exposure time, which suggests the occurrence of MIC. The *I_corr_* value was highest in the presence of *D. desulfuricans*. Furthermore, the current density of the anodic branch in the 304SS coupons in the SRB solution was higher than that of those immersed in blank solutions for each corresponding immersion time. These observations can be attributed to the aggressive role of bacteria in enhancing the corrosion of stainless steel by destroying the oxide layer on stainless steel [54]. The *I_corr_* value and current density of the anodic branch for the 304SS coupons immersed in the BDMDAC solution significantly decreased compared with coupons after 14 and 28 days of immersion in the SRB solution. These results indicate the ability of BDMDAC to protect stainless steel substrate against *D. desulfuricans*. Notably, the potentiodynamic polarization curves of coupons exposed to the BDMDAC solution still exhibited a little positive shift compared with those of the blank solution curves. This observation can be attributed to the fact that the bacterial adhesion and biofilm formation at very beginning of immersion test could affect the physical and chemical conditions of 304SS coupons, and result in adverse effects on their corrosion performance. Figure 6 shows the OM images of 14-day immersed coupons after potentiodynamic polarization curve measurements. As can be seen, the obvious localized corrosion was found on the 304SS coupon in the SRB solution (as indicated by red arrows in the Figure 6b). This can be attributed to the MIC effect, leading to the deterioration of passive film by sulfide produced by SRB [42] after being immersed in the SRB solution. These results provide considerable support for our potentiodynamic polarization curve results that the lower current density of the anodic branch and poor passivity were found on the 304SS coupon immersed in the SRB solution.

### 3.5. Limitations of This Study

Electrochemical techniques include potentiodynamic polarization are useful for examining the corrosion performance of metallic substrates. However, some limitations of electrochemical techniques for investigating microbial corrosion have been proposed. Javaherdashti has mentioned that large polarizations could be deleterious to micro-organisms in the biofilm [55]. However, it should be noticed that the two examples provided in this reference focused on cathodic protection and its effect(s) on MIC. A possible mechanism is that the application of cathodic potential will increase local pH (due to ORR reaction: O_2_ + 2H_2_O + 4e^−^ → 4OH^−^) and result in inhibiting the bacterial reproduction of microbes in such a high alkaline environment [56,57]. In addition, the time staying in the cathodic polarization potential is another important issue to study the effects of cathodic polarization on MIC. Little et al. [58] and Romero et al. [59] had polarized their samples at –400 mV (vs. SCE) for 72 h and –1000 mV (vs. SCE) for 3–72 h, respectively, to investigate the cathodic polarization effects on MIC. In contrast, the length of sample staying in the cathodic polarization potential is only 500 s (from –500 mV to 0 mV vs. open circuit potential (OCP), scan rate: 1 mV/s) in the present study. As a result, it is believed that the electrochemical experimental setup used in this study is likely to have less influence on the microbial environment.

On the other hand, potentiodynamic polarization curves were not periodically made during the immersion test in the present study. These measurements were applied and started applying potential on the 304SS coupons only after 14 or 28 days of immersion. Although the bacterial activity may be influenced during the polarization test, MIC-induced changes in the electrochemical conditions of 304SS coupons during 14 or 28 days of immersion had resulted in adverse effects on their corrosion performance. The potentiodynamic polarization curve measurement of coupons under different corrosion conditions (with and without bacteria) after different exposure times had also been used in numerous relevant studies [5,42,43,44]. In addition, the coupons for 14- and 28-day immersion tests were prepared in the different bottles, indicating the polarization potential applied on the 14-day immersed coupons would not have influence on the bacteria and culture medium in 28-day immersed ones. For all the above reasons, the electrochemical analysis used in this study gives useful information for research purposes.

## 4. Conclusions

In this study, potentiodynamic polarization curves were employed together with SEM and CLSM techniques to investigate corrosion and microbial corrosion inhibition of BDMDAC for 304SS substrates. The results reveal that BDMDAC achieved satisfactory results against microbial corrosion by *D. desulfuricans* in anaerobic conditions. Addition of 25 ppm BDMDAC could effectively inhibit the growth of *D. desulfuricans*. Surface characterization results revealed no biofilm on the 304SS coupons surfaces after BDMDAC was added to the *D. desulfuricans*-inoculated medium. The electrochemical results revealed deceleration of corrosion caused by the addition of BDMDAC in the *D. desulfuricans*-inoculated medium, which was demonstrated by the decrease of current densities in the anodic region and *I_corr_*.

## Figures and Tables

**Table 1:** EDS analysis results after 14 and 28 days of immersion.

Immersion Times	Elements (wt %)	O	S	Cr	Fe	Ni	Si
14 days	Spectrum 1	8.39	4.67	14.66	64.57	5.42	2.29
28 days	Spectrum 2	-	-	19.59	71.51	8.23	0.67
	Spectrum 3	32.26	14.34	-	53.40	-	-
	Spectrum 4	-	-	19.36	72.53	8.11	-

**Table 2:** Corrosion potential and corrosion current density measured according to potentiodynamic polarization curves after 14 and 28 days of immersion.

Samples	Blank 14 days	SRB 14 days	BDMDAC 14 days	Blank 28 days	SRB 28 days	BDMDAC 28 days
*E_corr_* (mV vs. SCE)	−755 ± 36	−733 ± 38	−758 ± 58	−751 ± 24	−765 ± 23	−764 ± 32
*I_corr_* (μA/cm^2^)	1.06 ± 0.06	2.39 ± 0.25	1.57 ± 0.02	0.95 ± 0.05	3.06 ± 0.25	1.83 ± 0.08
